# A Systematic Review of Statistical Methods Used to Test for Reliability of Medical Instruments Measuring Continuous Variables

**Published:** 2013-06

**Authors:** Rafdzah Zaki, Awang Bulgiba, Noorhaire Nordin, Noor Azina Ismail

**Affiliations:** 1 *Julius Centre University of Malaya, Department of Social & Preventive Medicine, Faculty of Medicine, University of Malaya, 50603, Kuala Lumpur, Malaysia*; 2 *Department of Applied Statistics, Faculty of Economics & Administration, University of Malaya, 50603, Kuala Lumpur, Malaysia*

**Keywords:** ICC, Intra-class correlation coefficient, Reliability, Statistical method, Validation study

## Abstract

***Objective(s):*** Reliability measures precision or the extent to which test results can be replicated. This is the first ever systematic review to identify statistical methods used to measure reliability of equipment measuring continuous variables. This studyalso aims to highlight the inappropriate statistical method used in the reliability analysis and its implication in the medical practice.

***Materials and Methods:*** In 2010, five electronic databases were searched between 2007 and 2009 to look for reliability studies. A total of 5,795 titles were initially identified. Only 282 titles were potentially related, and finally 42 fitted the inclusion criteria.

***Results:*** The Intra-class Correlation Coefficient (ICC) is the most popular method with 25 (60%) studies having used this method followed by the comparing means (8 or 19%). Out of 25 studies using the ICC, only 7 (28%) reported the confidence intervals and types of ICC used. Most studies (71%) also tested the agreement of instruments.

***Conclusion:*** This study finds that the Intra-class Correlation Coefficient is the most popular method used to assess the reliability of medical instruments measuring continuous outcomes. There are also inappropriate applications and interpretations of statistical methods in some studies. It is important for medical researchers to be aware of this issue, and be able to correctly perform analysis in reliability studies.

## Introduction

Reliability is one of the important parameters in determining the quality of an instrument. Reliability measures precision or the extent to which test results can be replicated (1). Many important variables measured in medicine are numerical or continuous in nature for example blood pressure, hemoglobin level, and oxygen level. Various statistical methods have been used to test the reliability of medical instruments with quantitative or continuous outcomes (2, 3). However, correlation coefficient (r), comparing means and Bland-Altman Limits of Agreement have been shown to be inappropriate for assessing reliability (2, 4). This study aims to identify statistical methods used to assess the reliability of medical instruments measuring continuous variables in the medical literature. This review also aims to highlight the inappropriate statistical method used in the reliability analysis and its implication in the medical practice. The proportion of various statistical methods found in this review will also reflect the level of knowledge (as determined by the statistical tests used) on the analysis of reliability.

## Materials and Methods

This review follows the standards as suggested in the PRISMA statement (5). 

***Searching***

*Inclusion criteria*

Only method comparison studies assessing the reliability of medical instruments measuring continuous variables, and applicable for use in humans will be considered. 

*Search strategy for identification of studies*

In 2010, we searched Medline (6), Ovid (7) , PubMed (   8), Scopus (9) and Science Direct (10) for studies investigating the reliability of instruments or equipment in medicine published in journals between January 2007 and December 2009. Boolean search was performed on each database using the search term: Reliability AND (validation OR “comparison study”).

The search was limited to the medical field (including dentistry), studies involving human subjects, and articles in the English language. Unpublished articles were not considered in this review.

***Study selection***

All citations identified from the search were downloaded into the EndNote X1 software. The citations were organized, and duplicates were identified and deleted. Two independent reviewers conducted the study selection. Any studies with qualitative or categorical data, studies with different units of outcomes, and association studies were excluded. There was no disagreement between the two reviewers at the stage of study selection. 

***Data extraction***

Information on the year of publication and statistical method used were extracted from each article. The statistical methods used to assess reliability were identified from the methodology section or the statistical analysis section, and by identifying which statistical methods influenced the author’s conclusion on the reliability of the instrument. The application of only correlation coefficient (r), comparing means and/or Bland-Altman Limits of Agreement were considered as inappropriate for reliability analysis. Two independent reviewers performed the data extraction. Most of the time, the spacing reviewers agreed with the outcomes. Only at two occasions there were some disagreement in deciding whether multiple or single statistical method was used to determine reliability. However, agreement was reached by consensus and assisted by a third reviewer. 

***Data analysis***

Descriptive analysis of the characteristics of studies and statistical methods used were performed. This is a descriptive review, and all results are displayed as percentages. Data analyses were performed using the SPSS 15.0 software. 

## Results

**Figure 1:**
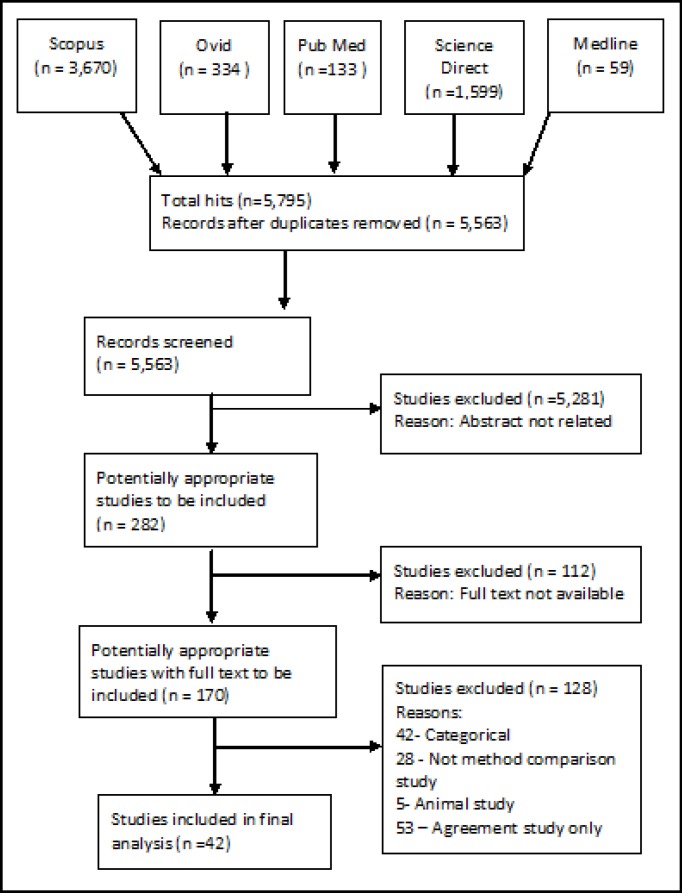
Flowchart of studies

A total of 5,795 titles were initially identified. However, after filtering for duplicates 5,563 titles and abstracts were reviewed. Only 282 were potentially related articles. A total of 170 full-text articles were reviewed. Of these, 131 articles did not meet the inclusion criteria, and a total of 42 articles were finally included in this review. Figure 1 summarizes the selection process. 

Out of the 42 articles reviewed, 26 (62%) were published in 2007, 7 (17%) in 2008, and 9 (21%) in 2009. Twelve (29%) articles were obtained from the Scopus database, 12 (29%) from the Science Direct database, 7 (17%) from the PubMed database, 6 (14%) from the Ovid and 5 (12%) from the Medline. 

Most of the reviewed studies (36 or 86%) relied on a single method to assess reliability. Others have used a combination of two or more methods. The Intra-class Correlation Coefficient (ICC) was the most popular method used to assess reliability and was used in 25 (60%) of the reviewed studies. This was followed by the comparing means (8 or 19%), Bland-Altman Limits of Agreement (7 or 17%), and correlation coefficient (2 or 5%). These findings are shown in Table 1. Thirty studies (71%) also measured agreement at the same time. Out of 25 studies using the ICC, only 7 (28%) studies reported the confidence intervals and types of ICC used. 

Out of seven studies that used the Bland-Altman Limits of Agreement, four studies (-) used only the Band-Altman Limits of Agreement to measure reliability, two studies (15, 16) used a combination with the ICC, and one study (17) used a combination with the correlation coefficient (r). Total of two studies used correlation coefficient (r) to determine reliability found in this review. Out of this two, one study (17) used a combination of correlation coefficient (r) and the Bland-Altman Limits of Agreement, and another study (18) used only the correlation coefficient to conclude on the reliability of tested instrument. Two (5%) studies (19, 20) have measured reliability using only the standard deviation of mean difference. 

**Table 1:** Most popular statistical methods used to assess reliability in medicine

Statistical method used	Number of methods used according to year of publication			
1. Intra-class correlation coefficient 2. Compare means/ mean difference 3. Bland- Altman limits of agreement 4. Correlation coefficient	2007 6 5 5 2	2008 5 2 0 0	2009 14 1 2 0	Total 25 8 7 2
				

## Discussion

This is the first systematic review on statistical methods used in reliability studies in the medical field. This study provides evidence that the Intra-class Correlation Coefficient (ICC) is the most popular method that has been used to measure reliability. Our study also shows that there were inappropriate applications of statistical methods to assess reliability in the medical literature. 

This study found that eight (19%) of reviewed articles have used inappropriate applications of statistical methods to assess reliability. The equipment which has been tested using these methods may not be reliable. It makes uncomfortable reading that as many as two out of ten supposedly reliable instruments currently used in medical practice may not be reliable. This has the potential to affect the management of patients, quality of care given to the patient, and worse still could cost lives.

*Intra-class correlation coefficient*

The Intra-class Correlation Coefficient was originally proposed by Ronald Fisher, in 1954(15)(14)(10)(9). 

The earliest ICCs were modifications of the Pearson Correlation Coefficient (22). There is no ordering of the repeated measures in the ICC, and it can be applied to more than two repeated measurements. However, the modern version of ICC is now calculated using variance estimates, obtained from the analysis of variance (ANOVA), through partitioning of the total variance between and within subject variance (2, 22).

ICC is a ratio of variances derived from ANOVA, so it is unit-less. The closer this ratio is to 1.0, the higher the reliability (22). Rosner (24) suggested that ICC < 0.4 indicated poor reliability, 0.4 ≤ ICC < 0.75 as fair to good reliability, and ICC ≥ 0.75 as excellent reliability. However, it must be remembered that the ICC is a point estimate and the interpretation is incomplete without the confidence interval. Out of the 25 studies that have used the ICC, only 7 (28%) studies reported the confidence intervals. Therefore the interpretations of ICC from the other 18 studies (72%) were questionable. 

There are different types of ICC. Weir (22) summarized different types of ICC, based on models introduced by Shrout and Fleiss (25), and McGraw and Wong (26). Weir have suggested certain factors need to be considered when choosing the type of ICC. This includes one- or two-way model, fixed- or random-effect model, and single or average measures (22). There has been considerable debate regarding the most appropriate type of ICC to be used in measuring reliability (27). Researcher are sometimes confused and unsure which type of ICC to use (22). Muller and Buttner (28) demonstrated that the different types of ICC may result in quite different values for the same dataset, under the same sampling theory. Therefore it is important to report the type of ICC that has been used in a reliability study. However, only 7 (28%) studies in this review reported the types of ICC used. 

*Means comparison*

The second most popular method found in this review is to compare means of two sets of measurements (either using the t-test or looking at the mean difference). However, the t-test only gives information about differences between the means of two sets of data, and not about individual differences (2). Hypothetical data (Table 2) of repeated readings for systolic blood pressure (SBP) of ten patients using an automatic blood pressure machine can demonstrate this.

The mean difference and standard deviation for the data from Table 2 are 5mmHg and 9mmHg. Paired t-test analysis of the data result in P=0.083 (i.e. no significant difference) which would indicate that the instrument is reliable. However, the data in Table 2 reflects an instrument with poor reliability. Bruton *et al*. (2) proposed that this method should not be used in isolation to assess reliability.

*Bland-Altman limits of agreement*

Another method that has been used to assess reliability found in this review is the Bland-Altman Limits of Agreement (LoA). This method was originally proposed for the analysis of agreement (3). Bland and Altman (29) suggested that LoA was also suitable for the analysis of repeatability of a single measurement method. 

**Table 2.** Hypothetical data on repeated reading of Systolic Blood Pressure (SBP)

Patient	SBP reading 1 (mmHg)	SBP reading 2 (mmHg)	Difference
1 2 3 4 5 6 7 8 9 10	110 112 114 114 119 123 124 128 134 135	100 104 116 118 124 133 134 140 148 149	-10 -8 2 4 5 10 10 12 14 14

However, the LoA is not suitable to evaluate reliability. It only estimates reliability when there are

two observations for each subject. This breaches the concept of reliability that allows unlimited repeated numbers of observations per subject (30). Although Bland and Altman (31) suggested methods to deal with multiple measurements in calculating the LoA, this method is more suitable for the analysis of agreement rather than reliability. 

The use of LoA in the analysis of reliability has also been criticised by Hopkins (2000). According to Hopkins (4), the values of the LoA can result in up to 21% bias depending on the degrees of freedom of the reliability study (i.e. number of participants and trials). Furthermore, Hopkins (4) added that LoA cannot be applied to the simplest situation of only one trial (e.g. a urine test for a banned substance in an athlete). 

*Correlation coefficient*

The correlation coefficient (r) has also been used to assess reliability. However, this method is also not suitable for the analysis of reliability. The correlation coefficient only provides information about the association and the strength of linear relationship. Correlation will not detect any systematic or fixed errors, and it is possible to have two sets of scores that are highly correlated, but not repeatable (2). The correlation coefficient for the data in Table 2 is 0.9757 (i.e. very high). However, it is clear that the values of second measurement were not even close to the values obtained from the first measurement. Therefore, it is recommended that the correlation coefficient should not be used in isolation for measuring reliability (2, 32). Furthermore, the correlation coefficient also breaches the concept of reliability as it only estimates reliability when there are only two observations for each subject (30). 

*Agreement*

Another important aspect in testing the quality of medical instruments is agreement. Agreement represents lack of measurement error. It assesses how close the results of repeated measurements are to the “true value” or the criterion value (33). A precise instrument or instrument with good reliability, will not necessarily measure the “true” value. Therefore, a reliable instrument is useless without knowing its accuracy. Ideally both reliability and agreement should be assessed together. However this review shows that not all researchers reported both agreement and reliability of the instrument in a single study. Although there is the possibility of agreement and reliability studies being conducted separately for the same instrument. Recently, a systematic review on statistical methods used to assess agreement has been published (34). Out of 210 articles found, only 62 (30%) assessed the reliability of instrument. This review (34) also found that there were inappropriate application of statistical methods in the analysis of agreement. These reflect the lack of knowledge in validation study among medical researchers. 

Issues found in this review suggest that there is a need for education in analysis of validation studies. Recommendations on how to perform analysis in reliability studies is also needed. This is important because over time numerous new instruments continue to be developed with the aim of finding a cheaper, non-invasive and safer method to test patients. Introducing a formal training could be one of the solutions to improve knowledge in this area. Much medical research training is concentrated on clinical trial and observational studies. Education on validation studies (agreement and reliability studies) appears to be neglected. Further evaluation and direct measurement of gaps of knowledge in validation study is needed.

*Strength and limitation*

This systematic review has several strengths. This is the first study specifically designed to retrieve information on statistical methods used to test for reliability of medical instruments measuring the same continuous variable. A broad search term was used to capture the largest possible number of publications on this topic. We also tried to reduce bias by using two independent reviewers during the selection of articles and data extraction. However, the results of this study may have *limited* generalizability due to selection bias. This review was limited to five electronic databases (Medline, Ovid, PubMed, Science Direct and Scopus), limited to accessible full text articles, and articles published only in English. The search was only performed using online databases, and unpublished articles were not considered. However, these databases have a very wide coverage of published medical journals and most high quality and high impact journals are published in English.

## Conclusion

In conclusion, various statistical methods have been used to assess the reliability of medical instruments measuring continuous variables. This study found that the ICC is the most popular method that has been used. There were also some inappropriate applications and interpretation of statistical methods used to assess reliability found in this review. Educating medical researchers on method in validation study and clear recommendations and guidelines on how to perform the analysis will improve the quality of research in this area. 
